# Intake reminders are effective in enhancing adherence to direct oral anticoagulants in stroke patients: a randomised cross-over trial (MAAESTRO study)

**DOI:** 10.1007/s00415-023-12035-z

**Published:** 2023-10-13

**Authors:** Fine Dietrich, Alexandros A. Polymeris, Valerie Albert, Stefan T. Engelter, Kurt E. Hersberger, Sabine Schaedelin, Philippe A. Lyrer, Isabelle Arnet

**Affiliations:** 1 Pharmaceutical Care Research Group, Department of Pharmaceutical Sciences, University of Basel, Klingelbergstrasse 50, 4056 Basel, Switzerland; 2 Department of Neurology and Stroke Centre, University Hospital Basel and University of Basel, Petersgraben 4, 4051 Basel, Switzerland; 3 Neurology and Neurorehabilitation, University Department of Geriatric Medicine Felix Platter, University of Basel, Burgfelderstrasse 101, 4055 Basel, Switzerland; 4 Clinical Trial Unit, Department of Clinical Research, University Hospital Basel and University of Basel, Schanzenstrasse 55, 4056 Basel, Switzerland

**Keywords:** Ischaemic stroke, Direct oral anticoagulants, Atrial fibrillation, Stroke, Medication adherence

## Abstract

**Background:**

Direct oral anticoagulants (DOAC) effectively prevent recurrent ischaemic events in atrial fibrillation (AF) patients with recent stroke. However, excellent adherence to DOAC is mandatory to guarantee sufficient anticoagulation as the effect quickly subsides.

**Aim:**

To investigate the effect of intake reminders on adherence to DOAC.

**Methods:**

MAAESTRO was a randomised, cross-over study in DOAC-treated AF patients hospitalised for ischaemic stroke. Adherence was measured by electronic monitoring for 12 months. After an observational phase, patients were randomised to obtain an intake reminder either in the first or the second half of the subsequent 6-month interventional phase. The primary outcome was *100%-timing adherence*. Secondary outcomes were *100%-taking adherence*, and overall timing and taking adherence. We analysed adherence outcomes using McNemar’s test or mixed-effects logistic models.

**Results:**

Between January 2018 and March 2022, 130 stroke patients were included, of whom 42 dropped out before randomisation. Analysis was performed with 84 patients (mean age: 76.5 years, 39.3% women). A *100%-timing adherence* was observed in 10 patients who were using the reminder, and in zero patients without reminder (*p* = 0.002). The reminder significantly improved adherence to DOAC, with study participants having 2.7-fold increased odds to achieve an alternative threshold of *90%-timing adherence* (OR 2.65; 95% CI 1.05–6.69; *p* = 0.039). A similar effect was observed for *90%-taking adherence* (OR 3.06; 95% CI 1.20–7.80; *p* = 0.019). Overall timing and taking adherence increased significantly when using the reminder (OR 1.70; 95% CI 1.55–1.86, *p* < 0.01; and OR 1.67; 95% CI 1.52–1.84; *p* < 0.01).

**Conclusion:**

Intake reminders increased adherence to DOAC in patients with stroke attributable to atrial fibrillation.

**Trial registration:**

ClinicalTrials.gov: NCT03344146.

**Supplementary Information:**

The online version contains supplementary material available at 10.1007/s00415-023-12035-z.

## Introduction

Non-adherence to chronic treatments is common, and the consequences are severe [1–3]. In stroke patients with atrial fibrillation (AF), anticoagulants are essential to prevent thromboembolic events, such as ischaemic strokes. The mainstay of treatment for AF patients are direct oral anticoagulants (DOAC), which are recommended in preference over vitamin K antagonists and have largely replaced them [4]. A literature review of 18 studies shows that adherence to DOAC is mediocre, ranging between 38.0 and 99.7% [1]. Low perceived treatment benefits and the necessity of medication are associated with sub-optimal adherence [5]. Unlike other preventive treatments, DOAC must be taken strictly on time owing to their comparatively short half-lives ranging from 8 to 17 h [6]. However, routine methods for monitoring adequate anticoagulation are lacking. Therefore, the evaluation of adherence to DOAC might facilitate the evaluation of treatment efficacy.

The level of adherence to DOAC that is required for sufficient stroke prevention remains unclear. Few studies suggest adherence thresholds to maintain the therapeutic concentration of DOAC [2, 7]. Solla-Ruiz et al. observed increased thromboembolic events in patients who took less than 95% of DOAC doses compared to perfect adherers [2]. In another study, the lowest risk for cardiovascular events occurred in patients taking at least 90% of DOAC doses [7]. These results highlight the clinical relevance of optimising adherence to DOAC to ensure treatment effectiveness. However, the impact of individual adherence patterns, such as delayed intakes, on the anticoagulation level is unknown. Several methods are available to measure adherence that deliver different information [8]. Electronic monitoring is the "gold standard" to assess treatment implementation because the data obtained highly correlate with the actual intake behaviour [9, 10]. Electronic adherence data deliver the exact time of each medicine intake, which allows for a detailed analysis of omitted doses, delays and intake intervals [6].

Although several interventions to optimise medication adherence have been proposed, their effectiveness is ambiguous. Studies that investigate adherence interventions are often limited by small samples or the poor description of complex interventions [11]. Promising interventions are motivational interviewing, feedback on adherence monitoring data, and intake reminders [12, 13]. However, which adherence-enhancing intervention is appropriate for patients after a stroke remains unknown. Even mild ischaemic events can cause neurological or cognitive deficits that might compromise medication adherence [14]. Thus, stroke survivors might require particular support with medication administration. This study aimed to investigate the effect of an intake reminder on the adherence to DOAC of stroke survivors.

## Methods

### Study design

**Fig. 1:**
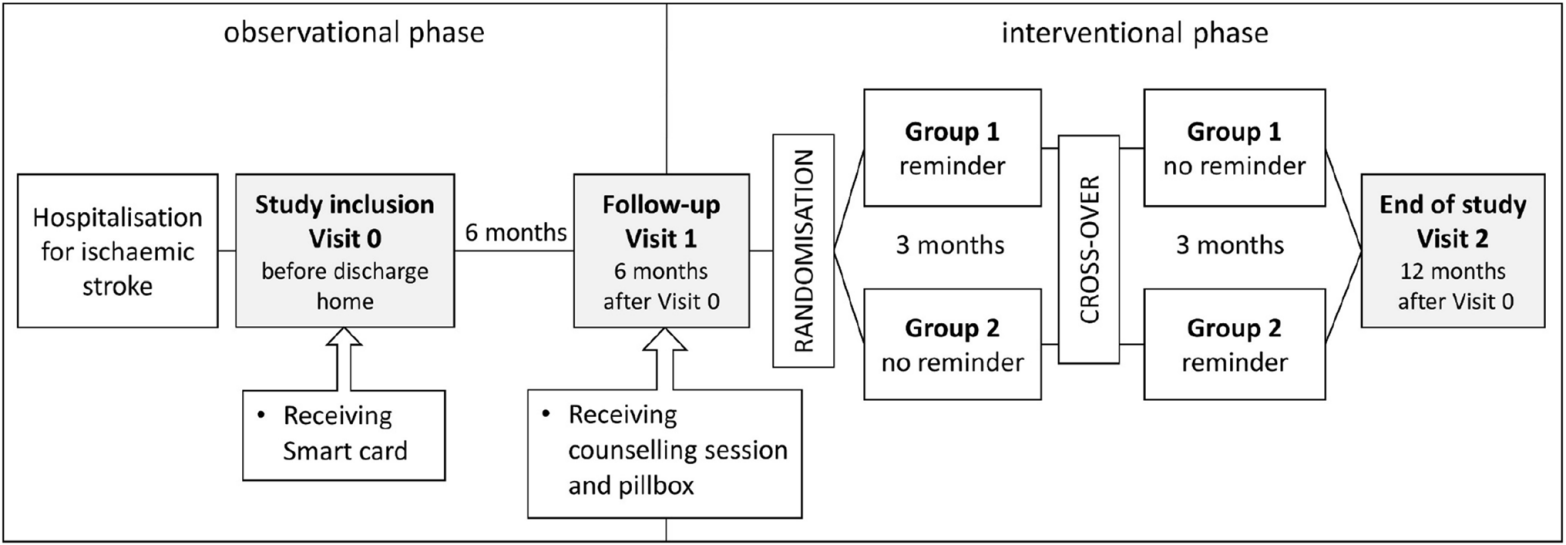
Flow chart of the MAAESTRO study [15]. At study inclusion (visit 0), patients received the Smart card to monitor DOAC intakes for 12 months. After six months (visit 1), all patients received a counselling session and a pillbox. According to randomisation, they received the reminder at visit 1 (group 1) or 3 months later (group 2), with a cross-over after 3 months

The MAAESTRO study (electronic **M**onitoring and improvement of **A**dherence to direct oral **A**nticoagulant treatment—a randomized crossover study of an **E**ducational and reminder-based intervention in ischemic **STRO**ke patients under polypharmacy) [15] included patients who (i) were hospitalised in the University Hospital of Basel, Switzerland, for ischaemic stroke, (ii) were diagnosed with AF, (iii) were prescribed a DOAC and at least two other medicines, and (iv) self-administered their medication. Recruitment of participants was performed during hospitalisation (visit 0). Participants electronically monitored their daily DOAC intakes for 12 months following hospital discharge using the Time4Med™ Smart card (Adherence Innovations, Hong Kong) [16]. By pushing the button of the Smart card, time stamps are recorded on a built-in microchip. The first 6 months after hospital discharge were observational. A staggered intervention was delivered. First, during the follow-up visit at 6 months (visit 1), a counselling session and a pillbox were provided to all patients. Then and according to randomisation, patients obtained an additional reminder device that was used beginning either at visit 1 (group 1) or 3 months later (group 2, Fig. 1), for a total of 3 months in each group. Study investigators were not involved in DOAC prescription, which was up to the hospital physician, general practitioners and other healthcare providers as standard of care.

Clinical and neuroimaging parameters (Fazekas score for white matter lesions on neuroimaging [17], National Institutes of Health Stroke Scale (NIHSS) for stroke severity [18], and Montreal Cognitive Assessment (MoCA) scores) [19] and self-reported variables (satisfaction with reminder) were assessed at each visit, as described in the study protocol [15].

### Study intervention

**Fig. 2:**
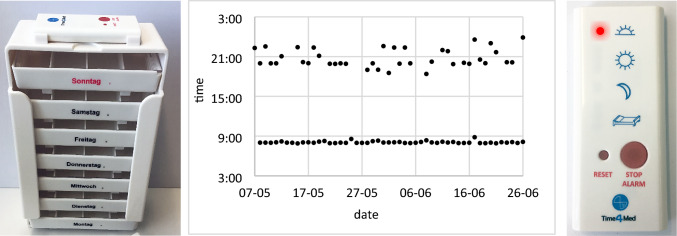
The main study intervention was a reminder that generates visible (red flashing light) and acoustic alarms (right). In addition, patients received a 7-day standard pillbox (left) and a counselling session based on the visualisation of the patient's monitoring data (black dots represent intake events) (middle)

The reminder was the main intervention in this trial. It generates acoustic and visual alarms at time points chosen by the patient. In addition, all patients received a pillbox and a counselling session with a pharmacist during which they were shown a visualisation of their intake data from the observational phase (Fig. 2).

### Study outcomes

The primary outcome was *100%-timing adherence*, defined as no dose taken outside the grace interval. The grace interval is defined as allowing an intake ± 6 h (for once-daily DOAC) and ± 3 h (for twice-daily DOAC) around the median intake time. We hypothesised that when patients use a reminder, they will reach *100%-timing adherence* more often than without a reminder.

The secondary outcomes were:

(i) percentage of patients with *100%-taking adherence* (no dose omitted),
(ii) overall timing adherence (proportion of doses taken within the grace interval, absolute value),
(iii) overall taking adherence (proportion of doses taken, absolute value),
(iv) occurrence of predefined clinical events of interest (recurrent ischaemic stroke, transient ischaemic attacks (TIA), intracranial haemorrhage, major extracranial haemorrhage, myocardial infarction, venous thromboembolism, death),
(v) patient satisfaction with the reminder (questionnaire, 5-point Likert-Scale).

### Data processing

We used secuTrial® (version 6.3.2.7) to capture electronic case report forms. The adherence monitoring data were cleaned, and metrics were calculated with Microsoft Excel® 2016 according to an internal standard operating procedure [20, 21]. Patients without sufficient electronic monitoring data records (less than one-third of the expected records was available) were excluded from the analysis.

### Sample size and randomisation

The sample size calculation was based on the following assumptions, informed by our previous experience with this patient population [15, 22]: (i) a 20% adherence-enhancing effect of the reminder on *100%-timing adherence*; and (ii) a 45% rate of *timing adherence* below 100% in the control group. The required sample size to achieve a power of 0.9 at an alpha error of 0.05 was 114 participants. In addition, a 12% drop-out rate was estimated. We therefore aimed to recruit 130 participants. Patients were randomly allocated to group 1 or group 2 in a 1:1 ratio. Randomisation was performed by a central computer during visit 1 over the web-based secuTrial® system with minimisation for sex, age, NIHSS and prior pillbox use at visit 0.

### Statistical analysis

The predefined statistical analysis plan dictated the use of mixed logistic models to assess the effect of reminders on *timing* and *taking adherence,* with reminder use ("with reminder" vs "without reminder") as fixed and subject identifier as a random effect. Due to the prohibitively small number of participants reaching the prespecified primary outcome of *100%-timing adherence*, we analysed the effect of reminders on the primary outcome using an exact McNemar’s test for paired nominal data instead and explored the alternative thresholds of *90%* and *80%-timing* and *taking adherence* using the planned mixed-effects analysis. For all analyses, we report two-sided *p*-values and present model-based odds ratio (OR) estimates with 95%-confidence intervals (CI).

Subgroup analyses were done by (i) DOAC type (once-daily vs twice daily), (ii) stroke location (right vs left hemisphere), (iii) Fazekas score (0–3) and (iv) MoCA score (0–30). Analyses were done on an intention-to-treat basis as prespecified in the statistical plan and were repeated in the per-protocol population (including only patients who used the reminder according to randomisation). We investigated time-trend and carry-over effects in sensitivity analyses. The effect of the counselling session and pillbox was assessed by comparing overall adherence during the observational phase and the first half of the interventional phase without the reminder (group 2). We present clinical events of interest and patient satisfaction with the reminder descriptively. All analyses were performed in R (version 4.1.2). Study outcomes were reported according to the CONSORT guideline [23].

### Ethics statement

The MAAESTRO study was approved by the Ethics Committee of Northwest/Central Switzerland (EKNZ 2017–01552) and registered at ClinicalTrials.gov (NCT03344146). All participants gave written informed consent; study participation was voluntary. For patients initially consenting to study participation but withdrawing their consent during the course of the study, the use of their study data up to the timepoint of consent withdrawal was allowed according to the signed informed consent. To accelerate the initiation of the interventional study phase, the study protocol was amended on April 28, 2020, to shorten the observational phase from 6 to 3 months for participants who entered the study after April 28, 2020.

## Results

### Population characteristics

**Fig. 3:**
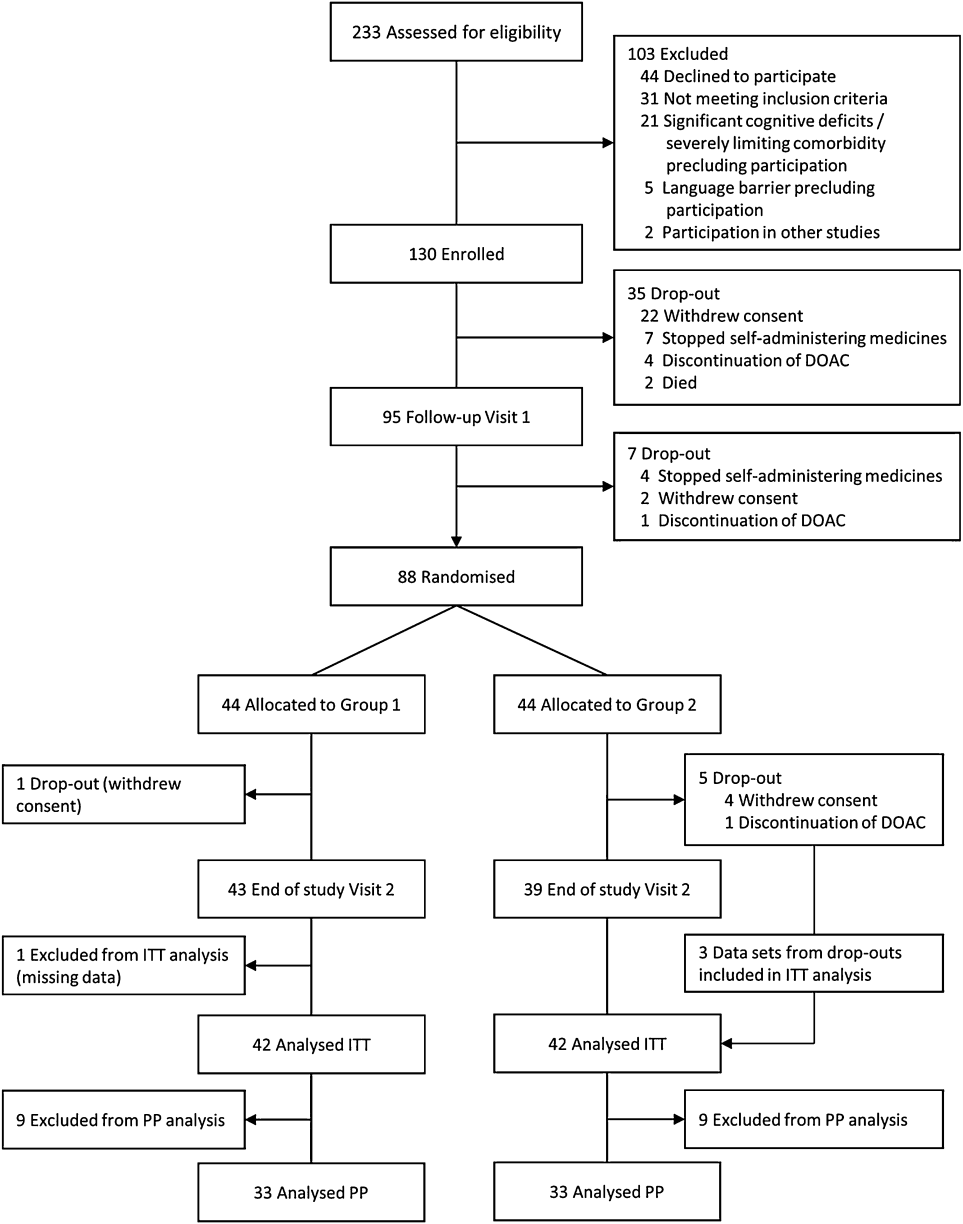
Flow diagram of MAAESTRO participants. *DOAC* direct oral anticoagulant, *ITT* intention-to-treat, *PP* per-protocol

From January 3, 2018, to March 14, 2022, 130 stroke patients were recruited, of whom 88 were randomised and 84 were eligible for analysis (Fig. 3). Mean [SD] age was 76.5 [9.1] years and 61% were male. Overall, stroke severity was mild (median [IQR] NIHSS 1 [0–2]). A twice-daily DOAC was prescribed in 74% of the patients and a once-daily DOAC in 26%. Participant characteristics were well-balanced between randomisation groups (Table 1; Supplement A).

**Table 1:** Characteristics of patients at study entry in the full analysis set per randomisation group

	Group 1	Group 2	SMD
*N*	42	42	
Age, years (mean (SD))	75.9 (8.5)	77.0 (9.8)	0.125
Female (%)	17 (40.5)	16 (38.1)	0.049
Comorbidities (%)			
Hypertension	36 (85.7)	37 (88.1)	0.071
Diabetes	5 (11.9)	12 (28.6)	0.424
Dyslipidemia	29 (69.0)	36 (85.7)	0.407
DOAC agent, at discharge (%)			0.169
Dabigatran	10 (23.8)	9 (21.4)	
Apixaban	20 (47.6)	23 (54.8)	
Rivaroxaban	7 (16.7)	5 (11.9)	
Edoxaban	5 (11.9)	5 (11.9)	
DOAC regimen, once-daily (%)	12 (30.0)	10 (23.8)	0.140
Days of hospitalisation (median [IQR])	7.5 [5.0–10.8]	5.0 [3.2–8.0]	0.500
Stroke hemisphere (%)			0.389
Left	16 (38.1)	14 (33.3)	
Right	11 (26.2)	16 (38.1)	
Both	10 (23.8)	5 (11.9)	
None	5 (11.9)	7 (16.6)	
Fazekas score (%)			0.104
1	18 (48.6)	20 (52.6)	
2	14 (37.8)	14 (36.8)	
3	5 (13.5)	4 (10.5)	
MoCA score (mean (SD))	23.9 (4.8)	24.5 (4.1)	0.133
mRS (median [IQR])	2.0 [1.0–2.0]	2.0 [1.0–2.0]	0.067
NIHSS (median [IQR])	1.0 [0.0–2.0]	1.0 [0.0–2.0]	0.145
Daily pill burden (median [IQR])	7.5 [5.0–9.8]	7.0 [5.0–9.8]	0.074
Prior pillbox use (%)	16 (38.1)	18 (42.8)	0.097
Prior use of anticoagulants (%)	18 (42.9)	19 (45.2)	0.048

*DOAC* direct oral anticoagulant, *IQR* interquartile range, *MoCA* Montreal Cognitive Assessment Score, *mRS* modified Rankin Scale, *NIHSS* National Institutes of Health Stroke Scale, *SD* standard deviation, *SMD* standardised mean difference

### Effect of reminders on adherence—intention-to-treat analysis

**Fig. 4:**
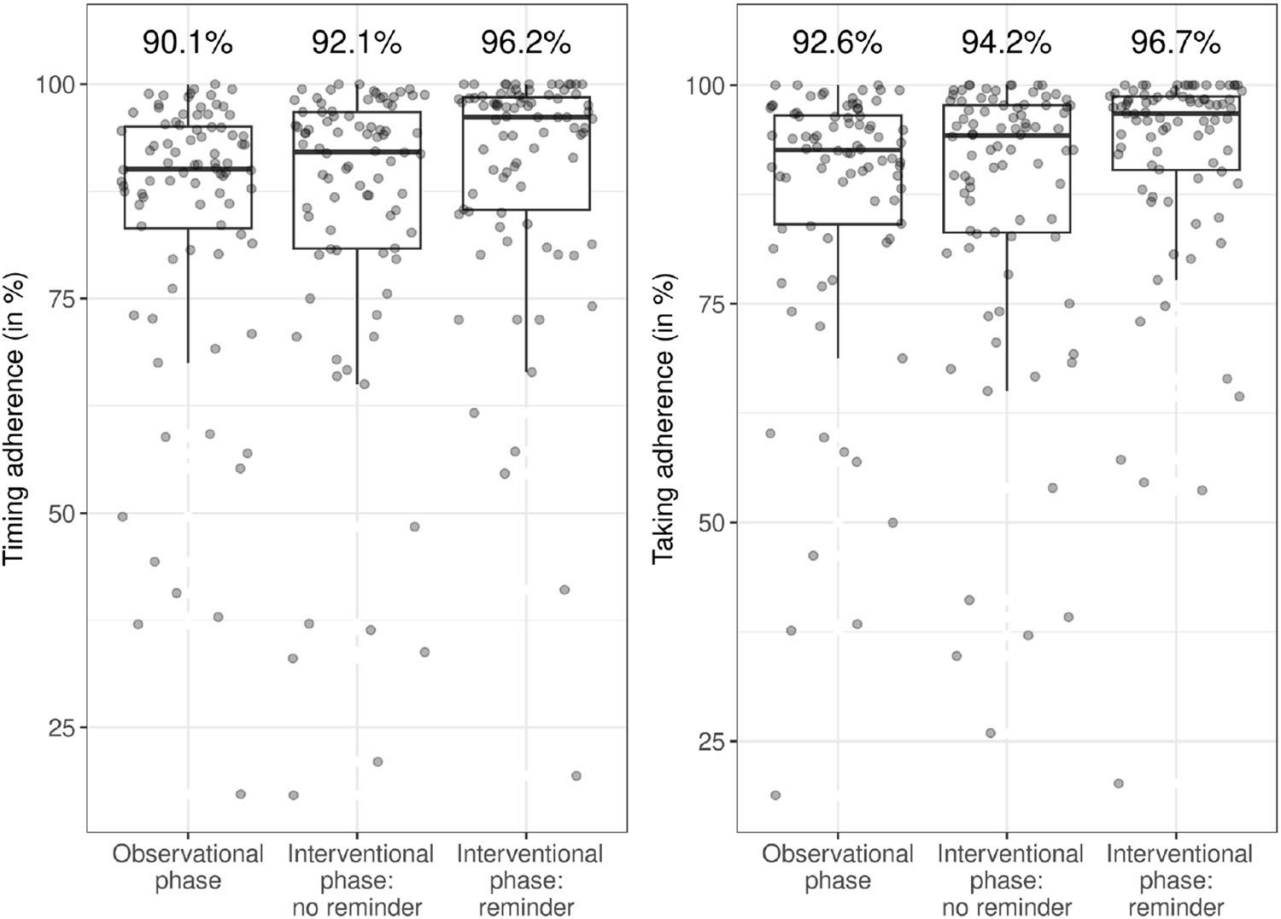
Boxplot of the median overall timing adherence (left) and taking adherence (right) of 84 participants per study phase

A *100%-timing adherence* was observed in 10 patients (12%) who were using the reminder, and in zero patients without the reminder (*p* = 0.002). A *100%-taking adherence* was observed in 10 patients (12%) who were using the reminder, and in 2 patients without (*p* = 0.039; Table 2). Patients reached *90%-timing adherence* (OR 2.65; 95% CI 1.05–6.69; *p* = 0.039) and *80%-timing adherence* (OR 25.9; 95% CI 1.51–444; *p* = 0.025) significantly more often with a reminder than without. With the reminder, the participants' overall timing adherence increased by 4% (median [IQR] 92% [81–97%] without reminders, 96% [85–99%] with reminders, Fig. 4). The odds of taking a DOAC dose on time increased by 70% with the use of a reminder (OR 1.7; 95% CI 1.55–1.86; *p* < 0.01).

**Table 2:** Number of patients (%) who reached different cut-offs of *timing adherence* and *taking adherence* with and without the use of the reminder (*N* = 84)

Adherence cut-offs	Never	Only without reminder	Only with reminder	Always	*p* value
*100%-timing adherence*	72 (85.7)	0 (0.0)	10 (11.9)	2 (2.4)	0.002
*90%-timing adherence*	21 (25.0)	5 (5.9)	15 (17.9)	43 (51.2)	0.041
*80%-timing adherence*	7 (8.3)	3 (3.6)	10 (11.9)	64 (76.2)	0.092
*100%-taking adherence*	68 (80.9)	2 (2.4)	10 (11.9)	4 (4.8)	0.039
*90%-taking adherence*	15 (17.9)	5 (5.9)	17 (20.2)	47 (55.9)	0.017
*80%-taking adherence*	6 (7.1)	3 (3.6)	10 (11.9)	65 (77.4)	0.092

In the last column, the results are compared using an exact McNemar’s test for paired nominal data

The use of the reminder increased the overall amount of DOAC doses taken, with significantly more patients reaching *90%-taking adherence* with the reminder than without (OR 3.06; 95% CI 1.20–7.80; *p* = 0.019). This also applied for *80%-taking adherence* (OR 27.4; 95% CI 1.56–480; *p* = 0.024). The overall taking adherence increased from a median [IQR] of 94% [83–98%] without reminders to 97% [90–99%] with reminders (Fig. 4). The odds of not missing a DOAC dose increased by 67% with the use of the reminder (Supplement B).

### Subgroup, per-protocol, and sensitivity analyses

The adherence-enhancing effect of reminders on *90%-timing adherence* did not differ in any of the predefined subgroups (Supplement C). The exclusion of 18 patients due to participants not using the reminder (9 participants per randomisation group) resulted in 66 participants eligible for the per-protocol analysis. Consistent with the intention-to-treat analysis, the effect of the reminder on *90%-timing adherence* was preserved by the per-protocol analysis (OR 2.53; 95% CI 0.86–7.41; *p* = 0.091). No carry-over effect (*p* = 0.950) or time-trend (*p* = 0.366) was observed between both interventional phases. There was no evidence that the delivery of a counselling session plus pillbox affected the number of patients reaching *90%-timing adherence* (Supplement C).

As a higher number of patients dropped out before randomisation, the evaluable sample in MAAESTRO fell short of the planned 114 full patient sets, despite the recruitment of the planned 130 patients. As a post hoc analysis, we calculated scenarios if our main outcome would have changed if 114 participants could have been included in the analysis. In the most likely scenario, our main outcome would remain unchanged (Supplement D.1).

### Clinical events of interest

A total of 21 clinical events of interest occurred throughout the study in 16 patients: recurrent ischaemic stroke (8), major extracranial haemorrhage (5), intracranial haemorrhage (4), myocardial infarction (2) and death (2). Fourteen clinical events occurred during the observational study phase (67%), five during the interventional phase without the reminder (24%), and two during the interventional phase with reminder (9%, see also Supplement D.2 and D.3).

A trend towards lower overall adherence was observed in patients with ischaemic events compared to patients without events (Table 3).

**Table 3:** Overall adherence during the observational study phase from all recruited participants, of whom 16 experienced clinical events of interest during the study

Group	Event	Timing adherence (%), median [IQR]	Taking adherence (%) median [IQR]
Any event^a^	Yes	90.7 [65.2–96.6]	91.6 [74.3–97.6]
	No	90.1 [83.5–94.9]	92.6 [86.8–96.4]
Haemorrhagic event	Yes	96.7 [92.9–98.4]	97.8 [93.7–98.6]
	No	89.9 [81.2–94.7]	92.3 [83.9–96.3]
Ischaemic event	Yes	73.5 [62.3–93.2]	76.3 [70.6–93.6]
	No	90.4 [83.5–95.3]	92.8 [86.8–96.4]

Haemorrhagic events include intracranial haemorrhage and major extracranial haemorrhage; ischaemic events include stroke, TIA, and myocardial infarction

*IQR* interquartile range, *TIA* transient ischaemic attack

^a^Two patients with clinical events, who died shortly after entering the study, are not included in this table because no adherence data were available.

### Satisfaction with the reminder

**Fig. 5:**
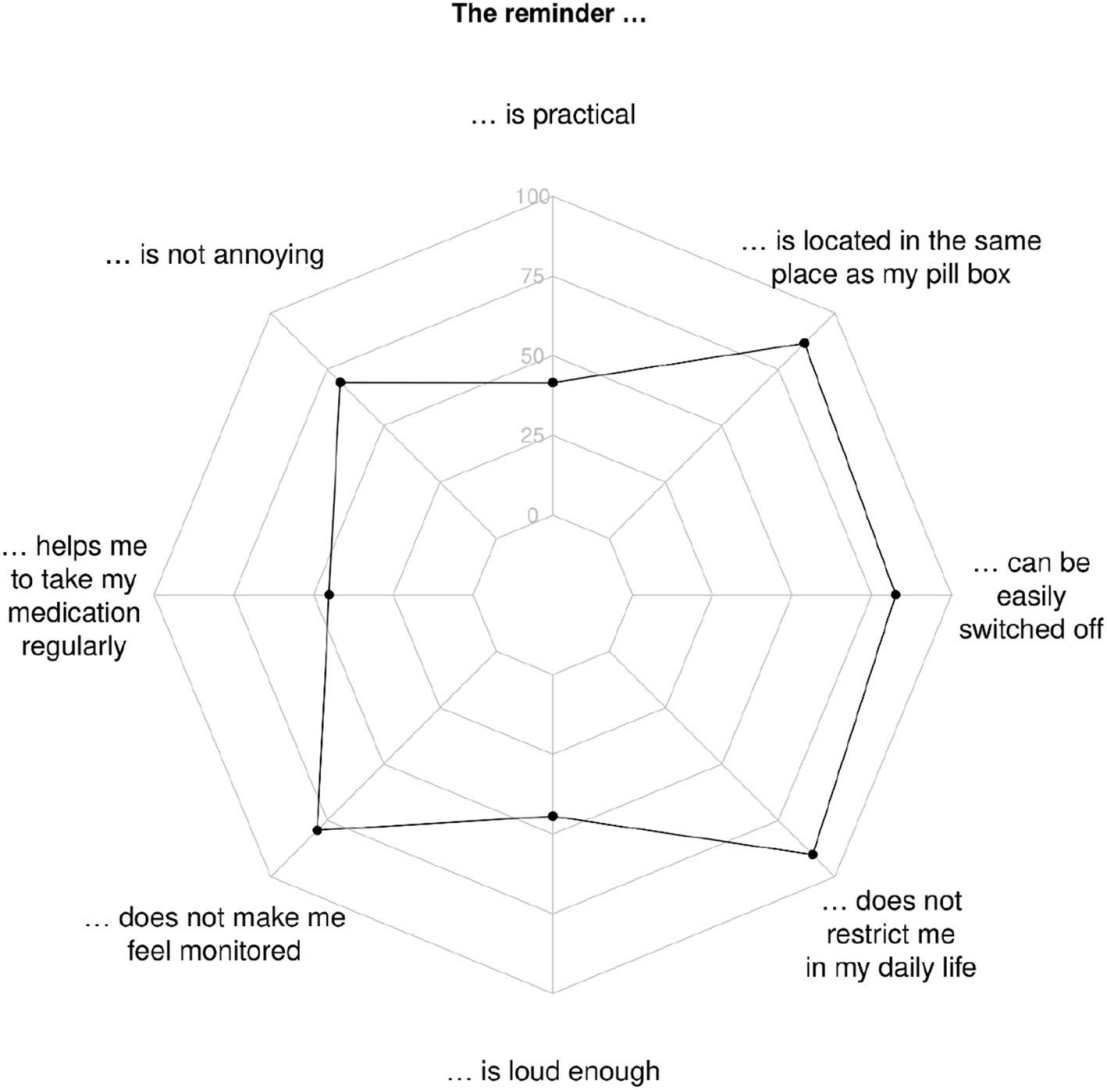
Answers to eight items regarding the satisfaction with the reminder from 84 patients. A value near the centre (0%) indicates poor agreement with the statement (see Supplement E for all values)

The satisfaction with the reminder differed considerably among participants (Fig. 5). About half of the participants found the reminder impractical and denied benefitting from it. Regarding the integration of the reminder into their daily routine, 81% of the participants felt no restriction (Supplement E).

## Discussion

The MAAESTRO study investigated the intake behaviour of patients with stroke attributable to atrial fibrillation, who are prescribed DOAC for the prevention of recurrent stroke. Specifically, we tested the effect of an intake reminder on adherence to DOAC. The primary outcome (*100%-timing adherence*) was almost solely achieved when participants used a reminder (*p* = 0.002). Although the numbers were too small for the mixed-model analysis (*N* = 10 with reminder, *N* = 0 without reminder), they are consistent with the findings from our alternative outcomes (*90%* and *80%-timing adherence*) and the Mc Nemar’s test where we found strong evidence that the use of reminders increase adherence.

The effect of reminders on adherence was shown in patients using long-term medication [13, 24], but the effectiveness of this approach in stroke patients in particular those with AF-related stroke has been unclear so far. The few studies that investigated the effect of reminders on adherence to DOAC have yielded discrepant results [25–29]. Some studies investigated only a single DOAC agent [28, 29] or they assessed adherence by self-report[26] or dispensing data[28]. These assessment methods are prone to manipulation by the patients [9], potentially explaining the discrepant findings of these studies. Notably, the behaviour of AF patients in general and not explicitly of stroke survivors was investigated in these previous studies. This is important because stroke-induced cognitive deficits might interfere with adherence. Therefore, our study contributes more detailed data on DOAC intake behaviours in the previously unexplored population of stroke survivors and confirms the effectiveness of reminders in this population.

Adherence to DOAC of the MAAESTRO population was already high at about 90%. Other studies that measured adherence to DOAC with electronic monitoring have reported comparable outcomes, although these studies did not specify if participants took DOAC for primary or secondary prevention, nor did they include all four DOAC agents [2, 29–31]. An a priori selection bias not amenable to randomisation procedures might explain these high adherence levels, with patients who opt to participate in an adherence study being more health-aware and thus potentially more prone to high adherence, a phenomenon termed "healthy-adherer effect" [32]. In addition, the MAAESTRO study was conducted in a comprehensive stroke centre with high expertise in stroke prevention and education. This might have resulted in overall higher adherence rates and limited the generalizability of our results.

Sensitivity analyses showed that the chronology in which patients used the reminder did not influence the adherence-enhancing effect of the reminder. In other words, patients who stopped the use of the reminder after 3 months (group 1) and returned to usual settings also returned to a lower level of adherence in the following 3 months. Thus, the adherence-enhancing effect did not persist when the reminder was removed, which is in agreement with previous findings [33]. Lee et al. investigated the effect of multicompartment punch cards and a pharmaceutical care service on adherence to polypharmacy in hypertensive patients. The intervention was highly effective and adherence increased significantly, but after removing the intervention, adherence returned almost to baseline values.

We investigated the clinical relevance of the study outcomes by means of descriptive statistics because our study was not powered to statistically analyse the effect of improved adherence on ischemic events. We observed that adherence to DOAC was not associated with the occurrence of any clinical vascular event or death. At least, higher adherence seems to be associated with numerically fewer ischemic events, including recurrent ischemic strokes. This observation is in line with other studies that have provided evidence of the positive impact of high adherence on less clinical events in AF patients [2, 7].

Because participants of the MAAESTRO study had experienced mild strokes, they might have presented with hidden dysfunctions that remained undetected with the scales commonly used in clinical practice, such as NIHSS. Nevertheless, patients' ability to manage their medication might be negatively affected by a stroke, independently of its severity [14]. Barrett et al. observed that stroke survivors overestimated their capability to self-administer medication shortly after the stroke, which was associated with memory deficits [34]. Therefore, we recommend adherence support to be offered also to patients who seemingly recover well from mild strokes. Providing adherence support could be realised in cooperation with community pharmacies [35].

### Strengths and limitations

This study has several strengths. First, the cross-over design of our study enabled each patient to serve as their own control. When investigating individual behaviours, this design delivers more robust results than inter-individual comparisons because the risk of confounders is negligible. We observed no time trends or carry-over effects between the cross-over phases, which supports the appropriateness of our design. Second, our study population was a well-described and homogeneous sample of patients treated with DOAC for secondary prevention due to a recent ischaemic stroke attributable to atrial fibrillation. Reminders increased adherence in this specific sample and should therefore be offered to these patients after suffering a stroke. Third, we used electronic monitoring to assess adherence, which is considered the gold standard when investigating the implementation of a medicine. The Smart cards delivered accurate information on intake times, which was crucial to judging the impact of reminders on timely DOAC intakes.

There were also limitations to this study. First, the primary outcome (*100%-timing adherence*) proved to be overambitious. When transposed into practice, this threshold means that one single delayed dose in 3 months suffices to disqualify for *100%-timing adherence*. Thus, the use of a theory-based approach to developing our study yielded an unrealisable goal for individuals with some active life. It is likely that applying realist research would have provided a different primary outcome. In any case, evidence on a more appropriate threshold for timing adherence to DOAC was limited in the literature. Hence, the alternative outcome of *90%-timing adherence* that we used in our secondary analysis is exploratory in nature. Second, the drop-out rate of 36% was considerably higher than the 12% that were estimated from the literature. This number must be put in light of the evolving character of a recent stroke. Drop-outs occurred mainly during the first weeks following study inclusion and concerned older participants with poorer cognitive and functional outcomes. Although this selection bias is difficult to prevent, it reduces the generalizability of our results. We assume that the recruitment timing was inadequate, as patients had just experienced a stroke, which can be considered a rather disturbing situation. Some patients might have overestimated their ability to pursue the study procedures in view of the unusual circumstances. In addition, some inclusion criteria for participation (e.g. self-administration of medication) were subject to changes after discharge, which increased the drop-out rate. However, the calculation of ‘worst case’ scenarios showed no change in the main outcome if the planned patient number would have completed the trial. Third, we experienced technical issues with the electronic devices. Participants regularly reported Smart cards that stopped beeping when registering the DOAC intake. We systematically documented these defects and excluded these periods from the calculation. Nevertheless, we cannot exclude that some adherence gaps were in reality device malfunctions.

## Conclusion

An intake reminder significantly increased timing adherence to DOAC and reduced the number of missed intakes in stroke patients. Offering reminders to stroke patients who self-administer their DOAC treatment represents a way to improve their adherence.

### Supplementary Information

Supplementary file1 (DOCX 37 KB)

## Data Availability

Data are available upon reasonable request.

## Declarations

### Guarantor

FD.

## References

[1] Raparelli V, Proietti M, Cangemi R (2017). Adherence to oral anticoagulant therapy in patients with atrial fibrillation. Focus on non-vitamin K antagonist oral anticoagulants. Thromb Haemost.

[2] Solla-Ruiz I, Villanueva-Benito I, Paredes-Galan E (2019). Differences between patient-driven adherence to vitamin K antagonists and direct oral anticoagulants. Do few missed doses matter? ACO-MEMS Study. Thromb Res.

[3] Tiili P, Leventis I, Kinnunen J (2021). Adherence to oral anticoagulation in ischemic stroke patients with atrial fibrillation. Ann Med.

[4] Hindricks G, Potpara T, Dagres N (2020). ESC Guidelines for the diagnosis and management of atrial fibrillation developed in collaboration with the European Association of Cardio-Thoracic Surgery (EACTS). Eur Heart J.

[5] Chambers JA, O'Carroll RE, Hamilton B (2011). Adherence to medication in stroke survivors: a qualitative comparison of low and high adherers. Br J Health Psychol.

[6] Vrijens B, Heidbuchel H (2015). Non-vitamin K antagonist oral anticoagulants: considerations on once- vs. twice-daily regimens and their potential impact on medication adherence. Europace.

[7] Kim D, Yang PS, Jang E (2020). The optimal drug adherence to maximize the efficacy and safety of non-vitamin K antagonist oral anticoagulant in real-world atrial fibrillation patients. Europace.

[8] Lehmann A, Aslani P, Ahmed R (2014). Assessing medication adherence: options to consider. Int J Clin Pharm.

[9] Vrijens B, Antoniou S, Burnier M (2017). Current situation of medication adherence in hypertension. Front Pharmacol.

[10] Anghel LA, Farcas AM, Oprean RN (2019). An overview of the common methods used to measure treatment adherence. Med Pharm Rep.

[11] Nieuwlaat R, Wilczynski N, Navarro T et al (2014) Interventions for enhancing medication adherence (Review). The Cochrane Libr 11:1–250 10.1002/14651858.CD000011.pub4 PMC7263418 25412402

[12] Demonceau J, Ruppar T, Kristanto P (2013). Identification and assessment of adherence-enhancing interventions in studies assessing medication adherence through electronically compiled drug dosing histories: a systematic literature review and meta-analysis. Drugs.

[13] Kini V, Ho PM (2018). Interventions to improve medication adherence: a review. JAMA.

[14] Terrill AL, Schwartz JK, Belagaje SR (2018). Best practices for the interdisciplinary rehabilitation team: a review of mental health issues in mild stroke survivors. Stroke Res Treat.

[15] Polymeris AA, Albert V, Hersberger KE (2018). Protocol for MAAESTRO: electronic monitoring and improvement of adherence to direct oral anticoagulant treatment-a randomized crossover study of an educational and reminder-based intervention in ischemic STROke patients under polypharmacy. Front Neurol.

[16] Arnet I, Rothen JP, Hersberger KE (2019). Validation of a novel electronic device for medication adherence monitoring of ambulatory patients. Pharmacy (Basel)..

[17] Fazekas F, Chawluk JB, Alavi A (1987). MR signal abnormalities at 1.5 T in alzheimer's dementia and normal aging. AJNR.

[18] Brott T, Adams HP, Olinger CP (1989). Measurements of acute cerebral infarction: a clinical examination scale. Stroke.

[19] Nasreddine ZS, Phillips NA, Bedirian V (2005). The Montreal Cognitive Assessment, MoCA: a brief screening tool for mild cognitive impairment. J Am Geriatr Soc.

[20] Albert V, Polymeris AA, Dietrich F (2020). Insights into direct oral anticoagulant therapy implementation of stroke survivors with atrial fibrillation in an ambulatory setting. J Stroke Cerebrovasc Dis.

[21] Dietrich F, Polymeris AA, Verbeek M (2021). Impact of the COVID-19 lockdown on the adherence of stroke patients to direct oral anticoagulants: a secondary analysis from the MAAESTRO study. J Neurol.

[22] Polymeris AA, Traenka C, Hert L (2016). Frequency and determinants of adherence to oral anticoagulants in stroke patients with atrial fibrillation in clinical practice. Eur Neurol.

[23] Schulz KF, Altman DG, Moher D (2010). CONSORT 2010 statement: updated guidelines for reporting parallel group randomised trials. BMJ.

[24] Tao D, Xie L, Wang T (2015). A meta-analysis of the use of electronic reminders for patient adherence to medication in chronic disease care. J Telemed Telecare.

[25] Labovitz DL, Shafner L, Reyes Gil M (2017). Using artificial intelligence to reduce the risk of nonadherence in patients on anticoagulation therapy. Stroke.

[26] Senoo K, Miki T, Ohkura T (2022). A smartphone app to improve oral anticoagulation adherence in patients with atrial fibrillation: prospective observational study. JMIR Mhealth Uhealth.

[27] Toscos T, Coupe A, Wagner S (2020). Engaging patients in atrial fibrillation management via digital health technology: the impact of tailored messaging. J Innov Card Rhythm Manag.

[28] Turakhia M, Sundaram V, Smith SN (2021). Efficacy of a centralized, blended electronic, and human intervention to improve direct oral anticoagulant adherence: Smartphones to improve rivaroxaban ADHEREnce in atrial fibrillation (SmartADHERE) a randomized clinical trial. Am Heart J.

[29] Montalescot G, Brotons C, Cosyns B (2020). Educational impact on apixaban adherence in atrial fibrillation (the AEGEAN STUDY): a randomized clinical Trial. Am J Cardiovasc Drugs.

[30] Desteghe L, Vijgen J, Koopman P (2018). Telemonitoring-based feedback improves adherence to non-vitamin K antagonist oral anticoagulants intake in patients with atrial fibrillation. Eur Heart J.

[31] Marquez-Contreras E, Martell-Claros N, Marquez-Rivero S (2018). Strategies for improving dabigatran adherence for stroke prevention in patients with non-valvular atrial fibrillation: education and drug intake reminders (FACILITA study). Curr Med Res Opin.

[32] Simpson SH, Eurich DT, Majumdar SR (2006). A meta-analysis of the association between adherence to drug therapy and mortality. BMJ.

[33] Lee JK, Grace KA, Taylor AJ (2006). Effect of a pharmacy care program on medication adherence and persistence, blood pressure, and low-density lipoprotein cholesterol a randomized controlled Trial. JAMA.

[34] Barrett AM, Galletta EE, Zhang J (2014). Stroke survivors over-estimate their medication self-administration (MSA) ability, predicting memory loss. Brain Inj.

[35] Farinha JM, Jones ID, Lip GYH (2022). Optimizing adherence and persistence to non-vitamin K antagonist oral anticoagulant therapy in atrial fibrillation. Eur Heart J Suppl.

