# Supplementary material for: Intake reminders are effective in enhancing adherence to direct oral anticoagulants in stroke patients: a randomised cross-over trial (MAAESTRO study)
Source: J Neurol. 2023 Oct 13;271(2):841–51. doi: 10.1007/s00415-023-12035-z (PMC10827905; doi:10.1007/s00415-023-12035-z)
Supplement: Supplementary file 1 — Supplementary file1 (DOCX 37 KB) [file 415_2023_12035_MOESM1_ESM.docx]

Supplementary material

**Supplement A**

**Table A** Characteristics of all recruited patients (N=130), sorted by included in the full analysis set (TRUE) and not included (FALSE)

|  | **FALSE** | **TRUE** | **P value** |
| --- | --- | --- | --- |
| N | 46 | 84 |  |
| Age, years (mean (SD)) | 79.5 (8.2) | 76.5 (9.1) | 0.063 |
| Female (%) | 21 (45.7) | 33 (39.3) | 0.604 |
| Comorbidities (%) |  |  |  |
| Hypertension | 40 (87.0) | 73 (86.9) | 1.000 |
| Diabetes | 9 (19.6) | 17 (20.2) | 1.000 |
| Dyslipidemia | 37 (80.4) | 65 (77.4) | 0.856 |
| DOAC agent, at discharge (%) |  |  | 0.928 |
| Dabigatran | 8 (18.2) | 19 (22.6) |  |
| Apixaban | 25 (56.8) | 43 (51.2) |  |
| Rivaroxaban | 6 (13.6) | 12 (14.3) |  |
| Edoxaban | 5 (11.4) | 10 (11.9) |  |
| DOAC regimen, once-daily (%) | 3 (6.5) | 22 (26.8) | <0.001 |
| Days of hospitalisation (median [IQR]) | 7.0 [4.0, 11.2] | 7.0 [4.0, 10.0] | 0.684 |
| Stroke hemisphere (%) |  |  | 0.638 |
| left | 12 (27.3) | 30 (35.7) |  |
| right | 13 (29.5) | 27 (32.1) |  |
| both | 11 (25.0) | 15 (17.9) |  |
| none | 8 (18.2) | 12 (14.3) |  |
| Fazekas score (%) |  |  | 0.339 |
| 1 | 21 (50.0) | 38 (50.7) |  |
| 2 | 12 (28.6) | 28 (37.3) |  |
| 3 | 9 (21.4) | 9 (12.0) |  |
| MoCA score (mean (SD)) | 20.9 (5.6) | 24.2 (4.5) | <0.001 |
| mRS (median [IQR]) | 2.5 [1.0, 3.8] | 2.0 [1.0, 2.0] | 0.001 |
| NIHSS (median [IQR]) | 2.0 [1.0, 3.0] | 1.0 [0.0, 2.0] | 0.016 |
| Daily pill burden (median [IQR]) | 7.5 [5.0, 10.0] | 7.0 [5.0, 10.0] | 0.908 |
| Prior pillbox use (%) | 20 (43.5) | 34 (40.5) | 0.884 |

DOAC: direct oral anticoagulant, IQR: interquartile range, MoCA: Montreal Cognitive Assessment Score, mRS: modified Rankin Scale, NIHSS: National Institutes of Health Stroke Scale, SD: standard deviation.

**Supplement B**

**Table B** Effect of the reminder on different adherence cut-offs and overall adherence.

| **Adherence outcome** | **OR** | **95%-CI** | **P value** |
| --- | --- | --- | --- |
| *90%-timing adherence* | 2.65 | [1.05,6.69] | 0.039 |
| *80%-timing adherence* | 25.9 | [1.51,444] | 0.025 |
| *90%-taking adherence* | 3.06 | [1.20,7.80] | 0.019 |
| *80%-taking adherence* | 27.4 | [1.56,480] | 0.024 |
| Overall timing adherence | 1.70 | [1.55,1.86] | *<*0.01 |
| Overall taking adherence | 1.67 | [1.52,1.84] | *<*0.01 |

**Supplement C**

**Table C.1** Effect of the reminder on patients reaching *90%-timing adherence* in four predefined subgroups.

| **Subgroups** |  | **OR** | **95% CI** | **p value** |
| --- | --- | --- | --- | --- |
| **DOAC type** | once-daily (N=22) vs  twice daily (N=60) | 3.02 | [0.30-30.8] | 0.350 |
| **Stroke location** | right (N=27) vs  left (N=30) hemisphere | 0.72 | [0.06-9.05] | 0.800 |
| **Fazekas score** | 2 (N=28) vs  1 (N=38) | 7.08 | [0.72-69.8] | 0.094 |
|  | 3 (N=9) vs  1 (N=38) | 2.50 | [0.12-50.6] | 0.551 |
| **MoCA score** | continuous range from 0 to 30 (N=84) | 1.04 | [0.85-1.27] | 0.680 |

DOAC: direct oral anticoagulant, MoCA: Montreal Cognitive Assessment

**Table C.2** The combined effect of the pillbox and counselling session on adherence (patients reaching *90%-timing adherence, 80%-timing adherence*, *90%-taking adherence* and *80%-taking adherence*) was assessed by comparing adherence during the observational phase to the first 3 months of the interventional phase without reminder (group 2).

| **Adherence outcome** | **OR** | **95%-CI** | **P value** |
| --- | --- | --- | --- |
| *90%-timing adherence* | 2.72 | [0.84,8.86] | 0.096 |
| *80%-timing adherence* | 27.17 | [1.76,419] | 0.018 |
| *90%-taking adherence* | 1.74 | [0.52,5.80] | 0.369 |
| *80%-taking adherence* | 11.4 | [1.05,122] | 0.045 |

**Table C.3** Adherence rates in the three study phases, separated by randomization group.

|  | **Study phase** | **mean** | **SD** | **median** | **Q1** | **Q3** |
| --- | --- | --- | --- | --- | --- | --- |
| **Timing adherence [%]** | |  |  |  |  |  |
| **All** | observational phase | 84.86 | 16.68 | 90.08 | 83.19 | 95.06 |
|  | interventional phase - no reminder | 85.15 | 18.52 | 92.12 | 80.84 | 96.75 |
|  | interventional phase - reminder | 90.15 | 14.03 | 96.15 | 85.33 | 98.47 |
| **Group 1** | observational phase | 85.95 | 14.81 | 90.26 | 83.46 | 96.22 |
|  | interventional phase - no reminder | 82.72 | 21.82 | 92.03 | 79.72 | 97.73 |
|  | interventional phase - reminder | 90.61 | 13.45 | 97.35 | 88.33 | 98.38 |
| **Group 2** | observational phase | 83.76 | 18.49 | 90.08 | 81.84 | 94.65 |
|  | interventional phase - no reminder | 87.57 | 14.35 | 93.08 | 85.67 | 95.16 |
|  | interventional phase - reminder | 89.70 | 14.75 | 94.61 | 85.21 | 98.56 |
| **Taking adherence [%]** | |  |  |  |  |  |
| **All** | observational phase | 87.02 | 15.93 | 92.58 | 84.08 | 96.53 |
|  | interventional phase - no reminder | 87.19 | 16.45 | 94.22 | 83.12 | 97.69 |
|  | interventional phase - reminder | 91.65 | 13.12 | 96.74 | 90.28 | 98.70 |
| **Group 1** | observational phase | 88.49 | 12.60 | 92.58 | 84.59 | 96.63 |
|  | interventional phase - no reminder | 85.38 | 18.33 | 93.70 | 82.73 | 98.32 |
|  | interventional phase - reminder | 92.38 | 11.64 | 97.75 | 90.97 | 98.73 |
| **Group 2** | observational phase | 85.55 | 18.71 | 92.45 | 84.81 | 96.45 |
|  | interventional phase - no reminder | 88.99 | 14.32 | 94.22 | 87.81 | 97.53 |
|  | interventional phase - reminder | 90.92 | 14.56 | 95.94 | 89.70 | 98.67 |

Q1: first quartile, Q3: third quartile, SD: standard deviation

**Supplement D**

**Table D.1** Number of patients who reached *100%-timing adherence* with and without the use of the reminder. ’Worst case’ scenarios how the main outcome could have changed if the planned number of 114 patients would have completed the study (that is 30 additional patients). In the second row, the most likely scenario of 4 additional disconcordant pairs is presented. If half of these patients performed in favor of ‘only without reminder’, shifting the odds from 0:10 (only without reminder vs only with reminder) to 2:12, the main conclusion of our study would still have been the same. The p-value indicates the result of the exact McNemar’s test.

| **Number of additional disconcordant pairs *** | **never** | **only with reminder** | **only without reminder** | **always** | **P value** |
| --- | --- | --- | --- | --- | --- |
| 3 | 99 | 11 | 2 | 2 | 0.022 |
| 4 | 98 | 12 | 2 | 2 | 0.013 |
| 5 | 97 | 12 | 3 | 2 | 0.035 |
| 6 | 96 | 13 | 3 | 2 | 0.021 |
| 7 | 95 | 13 | 4 | 2 | 0.049 |
| 8 | 94 | 14 | 4 | 2 | 0.031 |
| 9 | 93 | 15 | 4 | 2 | 0.019 |
| 10 | 92 | 15 | 5 | 2 | 0.041 |

*disconcordant pairs are patients with different results in the control vs the interventional phase

**Table D.2** Overview of clinical events, separated by type of event, study phase and randomization group

|  | **All** | **Randomization group** | | | **Study phase** | | |
| --- | --- | --- | --- | --- | --- | --- | --- |
| **Type of event** |  | **Group 1** | **Group 2** | **n.a.** | **OBS** | **INV - NR** | **INV - R** |
| **Heamorrhagic events** | **9** | **4** | **3** | **2** | **5** | **4** | **-** |
| Major extracranial haemorrhage | 5 | 2 | 2 | 1 | 3 | 2 | - |
| Intracranial haemorrhage | 4 | 2 | 1 | 1 | 2 | 2 | - |
| **Ischaemic events** | **10** | **5** | **3** | **2** | **7** | **1** | **2** |
| Recurrent ischaemic stroke | 8 | 4 | 2 | 2 | 6 | - | 2 |
| Myocardial infarction | 2 | 1 | 1 | - | 1 | 1 | - |
| **Death** | **2** | - | - | 2 | 2 | - | - |

n.a.: not applicable (participant left the study before randomization), INV – NR: interventional study phase no reminder, INV – R: interventional study phase with reminder, OBS: observational study phase

**Table D.3** Overview of patients with clinical events, including the study phase, type of event and randomization group

| **Patient Nr.** | **FAS** | **Randomization group** | **Study phase event no. 1** | **Type of event no. 1** | **Study phase event no. 2** | **Type of event no. 2** |
| --- | --- | --- | --- | --- | --- | --- |
| 1 | TRUE | Group 2 | OBS | Intracranial haemorrhage | - | - |
| 2 | TRUE | Group 1 | OBS | Recurrent ischaemic stroke | OBS | Recurrent ischaemic stroke |
| 3 | FALSE | - | OBS | Recurrent ischaemic stroke | OBS | Death |
| 4 | FALSE | - | OBS | Death | - | - |
| 5 | FALSE | - | OBS | Intracranial haemorrhage | OBS | Major extracranial haemorrhage |
| 6 | TRUE | Group 1 | OBS | Recurrent ischaemic stroke | OBS | Major extracranial haemorrhage |
| 7 | TRUE | Group 2 | OBS | Recurrent ischaemic stroke | - | - |
| 8 | TRUE | Group 2 | INV - NR | Myocardial infarction | - | - |
| 9 | TRUE | Group 1 | OBS | Myocardial infarction | - | - |
| 10 | TRUE | Group 2 | INV - NR | Major extracranial haemorrhage | - | - |
| 11 | TRUE | Group 2 | INR - R | Recurrent ischaemic stroke | - | - |
| 12 | TRUE | Group 2 | INV - NR | Major extracranial haemorrhage | - | - |
| 13 | TRUE | Group 1 | INV - R | Recurrent ischaemic stroke | - | - |
| 14 | TRUE | Group 1 | INV - NR | Intracranial haemorrhage | INV - NR | Intracranial haemorrhage |
| 15 | FALSE | - | OBS | Recurrent ischaemic stroke | - | - |
| 16 | TRUE | Group 1 | OBS | Major extracranial haemorrhage | - | - |

FAS: full analysis set, INV – NR: interventional study phase no reminder, INV – R: interventional study phase with reminder, OBS: observational study phase

**Supplement E**

**Table E** Answers to the reminder satisfaction questionnaire. Patients could answer on a 5 point Likert scale.

| **The reminder …** | **Answers** | **n** | **%** |
| --- | --- | --- | --- |
| … is practical | strongly disagree | 24 | 35.3 |
|  | somewhat disagree | 14 | 20.6 |
|  | somewhat agree | 9 | 13.2 |
|  | strongly agree | 18 | 26.5 |
|  | I don’t know | 2 | 2.9 |
|  | missing value | 1 | 1.5 |
| … is annoying | strongly disagree | 44 | 64.7 |
|  | somewhat disagree | 1 | 1.5 |
|  | somewhat agree | 6 | 8.8 |
|  | strongly agree | 14 | 20.6 |
|  | I don’t know | 2 | 2.9 |
|  | missing value | 1 | 1.5 |
| … helps me to take my medicines more regularly | strongly disagree | 30 | 44.1 |
|  | somewhat disagree | 4 | 5.9 |
|  | somewhat agree | 10 | 14.7 |
|  | strongly agree | 18 | 26.5 |
|  | I don’t know | 2 | 2.9 |
|  | missing value | 4 | 5.9 |
| … makes me feel monitored | strongly disagree | 48 | 70.6 |
|  | somewhat disagree | 2 | 2.9 |
|  | somewhat agree | 3 | 4.4 |
|  | strongly agree | 10 | 14.7 |
|  | I don’t know | 3 | 4.4 |
|  | missing value | 2 | 2.9 |
| … is not loud enough | strongly disagree | 26 | 38.2 |
|  | somewhat disagree | 2 | 2.9 |
|  | somewhat agree | 11 | 16.2 |
|  | strongly agree | 24 | 35.3 |
|  | I don’t know | 3 | 4.4 |
|  | missing value | 2 | 2.9 |
| … restricts me in my daily life | strongly disagree | 55 | 80.9 |
|  | somewhat disagree | 0 | 0.0 |
|  | somewhat agree | 1 | 1.5 |
|  | strongly agree | 5 | 7.4 |
|  | I don’t know | 4 | 5.9 |
|  | missing value | 3 | 4.4 |
| … meets my needs | strongly disagree | 32 | 47.1 |
|  | somewhat disagree | 8 | 11.8 |
|  | somewhat agree | 5 | 7.4 |
|  | strongly agree | 17 | 25.0 |
|  | I don’t know | 3 | 4.4 |
|  | missing value | 3 | 4.4 |
| … helps me not to forget my medicines | strongly disagree | 29 | 42.6 |
|  | somewhat disagree | 1 | 1.5 |
|  | somewhat agree | 6 | 8.8 |
|  | strongly agree | 22 | 32.4 |
|  | I don’t know | 6 | 8.8 |
|  | missing value | 4 | 5.9 |

*Table continues on the next page*

| **The reminder …** | **Answers** | **n** | **%** |
| --- | --- | --- | --- |
| … makes me feel safe | strongly disagree | 28 | 41.2 |
|  | somewhat disagree | 3 | 4.4 |
|  | somewhat agree | 9 | 13.2 |
|  | strongly agree | 18 | 26.5 |
|  | I don’t know | 6 | 8.8 |
|  | missing value | 4 | 5.9 |
| … can be easily switched off | strongly disagree | 5 | 7.4 |
|  | somewhat disagree | 5 | 7.4 |
|  | somewhat agree | 5 | 7.4 |
|  | strongly agree | 42 | 61.8 |
|  | I don’t know | 10 | 14.7 |
|  | missing value | 1 | 1.5 |
| … is located in a different place than my pillbox | strongly disagree | 57 | 83.8 |
|  | somewhat disagree | 1 | 1.5 |
|  | somewhat agree | 1 | 1.5 |
|  | strongly agree | 8 | 11.8 |
|  | I don’t know | 1 | 1.5 |
|  | missing value | 0 | 0.0 |
| … helps me to take my medicines on time | strongly disagree | 27 | 39.7 |
|  | somewhat disagree | 2 | 2.9 |
|  | somewhat agree | 6 | 8.8 |
|  | strongly agree | 24 | 35.3 |
|  | I don’t know | 6 | 8.8 |
|  | missing value | 3 | 4.4 |
| … helps with my fear of not taking my medicines correctly | strongly disagree | 40 | 58.8 |
|  | somewhat disagree | 2 | 2.9 |
|  | somewhat agree | 4 | 5.9 |
|  | strongly agree | 6 | 8.8 |
|  | I don’t know | 10 | 14.7 |
|  | missing value | 6 | 8.8 |
| … lets me manage my medicines more independently | strongly disagree | 32 | 47.1 |
|  | somewhat disagree | 2 | 2.9 |
|  | somewhat agree | 7 | 10.3 |
|  | strongly agree | 17 | 25.0 |
|  | I don’t know | 6 | 8.8 |
|  | missing value | 4 | 5.9 |
